# Enhancing Chain Mobility of Ultrahigh Molecular Weight Polyethylene by Regulating Residence Time under a Consecutive Elongational Flow for Improved Processability

**DOI:** 10.3390/polym13132192

**Published:** 2021-06-30

**Authors:** Xiaochuan Chen, Xiaotong Wang, Yanhong Feng, Jinping Qu, Dingshan Yu, Changlin Cao, Xudong Chen

**Affiliations:** 1 Key Laboratory for Polymeric Composite and Functional Materials of Ministry of Education, Key Laboratory of High Performance Polymer-Based Composites of Guangdong Province, School of Chemistry, Sun Yat-Sen University, Guangzhou 510275, China; chenxch29@mail2.sysu.edu.cn (X.C.); wangxt58@mail2.sysu.edu.cn (X.W.); 2 Key Laboratory of Polymer Processing Engineering of the Ministry of Education, National Engineering Research Center of Novel Equipment for Polymer Processing, South China University of Technology, Guangzhou 510641, China; yhfeng@scut.edu.cn (Y.F.); jpqu@scut.edu.cn (J.Q.); 3 Fujian Key Laboratory of Pollution Control & Resource Reuse, Engineering Research Center of Polymer Green Recycling of Ministry of Education, College of Environmental Science and Engineering, Fujian Normal University, Fuzhou 350007, China

**Keywords:** elongational flow, ultrahigh molecular weight polyethylene, entanglement, molecular orientation, diffusion

## Abstract

Improving the processability of ultrahigh molecular weight polyethylene (UHMWPE) and understanding the effect of the polymeric chain mobility has long been a challenging task. Herein, we show that UHMWPE without any processing aids can be processed at a lower temperature of 180 °C compared to conventional processing temperatures (~250 °C) under a continuous elongational flow (CEF) by using an eccentric rotor extruder (ERE). By probing the effect of the residence time of UHMWPE samples under a CEF on the morphology, rheological behavior and molecular orientation, we find that the long polymer chains of UHMWPE are apt to orientate under a consecutive volume elongational deformation, thereby leading to a higher residual stress for the extruded sample. Meanwhile, the residence time of samples can regulate the polymeric chain mobility, giving rise to the simultaneous decrease of the melting defects and residual stress as well as Hermans orientation function with increasing residence time from 0 to 60 s. This also engenders the enhanced diffusion of UHMWPE segments, resulting in a defect-free morphology and higher entanglement with lower crystallinity but without causing obvious thermal oxidative degradation of UHMWPE. This interesting result could originate from the fast chain entanglement and particle welding enabled by a desirably short residence time, which could be explained by the empirical, entropy-driven melting explosion mechanism.

## 1. Introduction

Ultrahigh molecular weight polyethylene (UHMWPE), a well-known high performance engineering thermoplastic polymer with a molecular weight greater than 1,000,000 g/mol exhibited excellent properties such as high toughness, abrasion resistance, and chemical resistance. UHMWPE has been extensively used in a range of industrial applications demanding wear resistance and toughness as well as low friction [1,2,3]. However, its ultralong chain and great entanglement density lead to the ultrahigh melt viscosity and inferior melt mobility that hinders the direct processing of UHMWPE by conventional extrusion or injection equipment. Meanwhile, other processing techniques such as compression molding and ram extrusion tend to suffer from the discontinuous forming process, low forming efficiency, and poor mixing homogenization. The low processability of UHMWPE via conventional processing techniques hampers the large-scale applications of UHMWPE materials [4]. To address these issues, various strategies have been developed to improve the processability of UHMWPE. Blending with a lower molecular weight polymer, such as polyethylene (PE), stearate, and polystyrene, is a commonly used method. However, the introduction of low molecular weight polymers often compromised the mechanical properties of UHMWPE composites, and the huge viscosity mismatch between the components gave rise to a poor morphology, thus requiring the exploration of complex processing techniques for better mixing morphology [5,6]. Furthermore, dissolving UHMWPE in a suitable solvent or using unconventional polymerization techniques/conditions (such as using a homogeneous single-site catalyst [7]) can lead to the disentanglement of UHMWPE chains. Although these methods have shown some advantages over conventional processing techniques, they cannot serve as a universal solution to meet the challenges of UHMWPE melt processing. As an emerging technique, high velocity compaction (HVC) [4,8,9] endows excellent UHMWPE processing capability and efficiency via sintering, and the accelerated chain diffusion could lead to a rather high ductility to welded interfaces of UHMWPE-nascent powders. However, the noncontinuity of HVC process and small products size have limited its large-scale practical applications in UHMWPE processing.

Recently, we applied a novel eccentric rotor extruder (ERE) to process UHMWPE without any processing aids [10]. The ERE technique, which can generate strong elongational flow to polymer melts by consecutive compression and stretching using a series continuous dynamical converging channels, exhibits the characteristics of forced positive displacement conveying an elongational flow field. The comparative results verify that the ERE technique could effectively reduce melting defects and yield better homogeneous morphology as compared to the conventional bath mixing based on a shear flow. More importantly, the extruding samples processed by the ERE showed improved retention of the viscosity average molecular weight of UHMWPE-nascent powders. However, due to the higher stress generated by the ERE and the prolonged relaxation time of UHMWPE chain, the residual stress in ERE-processed UHMWPE products would cause an unexpected post-processing deformation.

In this contribution, we demonstrate that UHMWPE without any processing aids can be processed at the temperature of 180 °C, far below conventional processing temperatures for UHMWPE (~250 °C), under a continuous elongational flow (CEF) by using an eccentric rotor extruder (ERE). In addition, we probe the effect of the residence time of UHMWPE in extruding stage under a CEF on the morphology, rheological behavior, and molecular orientation. We show that desirably varying residence time can modulate the chain mobility of UHMWPE, leading to simultaneous decrease of the melting defects, residual stress, as well as Herman’s orientation function while enhancing the diffusion of UHMWPE segments to enable a defect-free morphology and higher entanglement with lower crystallinity but without obvious molecular degradation. We also propose the diffusion mechanism of UHMWPE under a consecutive elongational flow.

## 2. Materials and Methods

Ultrahigh molecular weight polyethylene (UHMWPE)-nascent powder (Mη = 3.00 × 10^6^ g/mol) was supplied by Shanghai Lianle Chemical Technology Co. (Shanghai, China).

### 2.1. Sample Preparation

UHMWPE samples were extruded by ERE (developed by the South China University of Technology, Guangzhou, China, shown in Figure S1) at 180 °C with a rotation rate of 15 rpm [6,10]. In order to achieve different residence time of the UHMWPE samples in the die head, the rotation rate of ERE is set to 0 rpm, while the residence time varies from 0 to 60 s.

### 2.2. Characterization

Viscosity average molecular weight (M_η_) values were derived from viscosity tests on an Ubbelohde viscometer according to the standard specification of ASTM D4020-2005, which was further calulated based on the Mark–Houwink equation: M_η_ = 5.37 × 10^4^ (IV)^1.49^, where IV denotes the intrinsic viscosity.

Differential scanning calorimetry (DSC) analysis was carried out on a TA Q20 Instrument (New Castle, DE, USA). The adopted temperature and heat flow scales were calibrated with high-purity indium. As-obtained samples were firstly heated to 200 °C and subsequently annealed for 5 min, followed by cooling down to 30 °C and re-heating to 200 °C at a rate of 10 °C/min. The crystallinity of samples was derived following the literature-reported method [11,12].

Raman mapping and polarized Raman spectra were done on the confocal micro-Raman spectroscopy (Thermo Scientific DXR2xi, Waltham, MA, USA) excited by an Ar^+^ laser of 532 nm. The excitation light with a power of 10 mW was focused on the samples with a Lycar objective lens of 50× microscope objective. Polarized Raman spectra with polarized beam perpendicular and parallel to the flow direction were also recorded to examine the molecular chain orientation of samples.

Rheological measurements were carried out on a stress-controlled rheometer (TA DHR-2 instrument, New Castle, DE, USA) by using a parallel plate under nitrogen protection. Dynamic frequency sweep of samples was performed in the linear viscoelastic region at 200 °C with rotation rates ranging from 0.01 to 100 rad/s (0.5% strain).

Morphologies of samples were examined on a Hitachi-S4800 (Tokyo, Japan) scanning electron microscope (SEM). 

Attenuated total reflection Fourier transform infrared (ATR-FTIR) measurements were performed on a NICOLET-iS-10 infrared spectrometer (Waltham, MA, USA).

Density tests were performed by the buoyancy method according to Archimedes’ principle. The collected density values were compared with those derived from the DSC analysis in order to roughly determine the remaining porosity of the samples by taking 0.855 g/cm^3^ and 1.000 g/cm^3^ as the densities of the amorphous and crystalline phases, respectively.

## 3. Results and Discussions

### 3.1. Morphology

**Figure 1:**
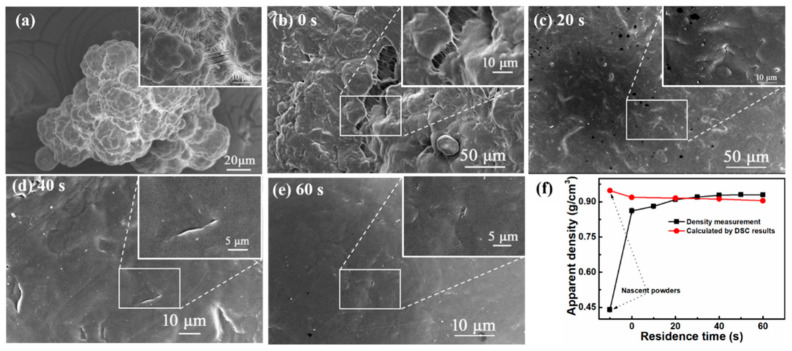
(**a**) SEM morphology of UHMWPE-nascent powders; (**b**–**e**) SEM morphology of the extruded UHMWPE sample with 0, 20, 40, and 60 s residence time, respectively; (**f**) the residence time varies from 0 to 60 s seconds density values determined from direct density measurement and calculated from DSC results.

As described in the Sample preparation section, UHMWPE samples are extruded at 180 °C with a rotation rate of 15 rpm by the ERE. The residence time of the UHMWPE samples in the die head of ERE varies from 0 to 60 s at a rotation rate of 0 rpm. SEM observation in Figure 1 displays the morphologies of the UHMWPE-nascent powder and the processed UHMWPE samples with different residence times ranging from 0 to 60 s under an elongational flow at 180 °C. The corresponding photographs of various samples are shown in Figure S2. Clearly, the UHMWPE-nascent powder consists of an agglomeration of small particles featuring a porous structure connected by a fibril network, as revealed by Figure 1a. In contrast, the extruded UHMWPE sample with the residence time of 0 s is out of shape (Figure S2), which implies a drastic residual stress retention. The SEM imaging with higher magnification in Figure 1b presents strip-like melting defects with exposed fibril-like structures caused by poor the thermal conductivity and high melting point of the UHMWPE-nascent powders. It is noted that the observed melting defects under an elongational flow is quite different from large cavity-like defects of UHMWPE samples produced under a shear flow at 180 °C. This result suggests that the UHMWPE-nascent powders are dramatically compressed by the ERE, since the elongational flow dominated in the ERE can induce a continuous elongational volumetric deformation for the UHMWPE-nascent powder, as described in our previous work [10]. With the increased residence time from 0 to 60 s, the post-process deformation of the extruded UHMWPE sample gradually decreases, owing to the increased relaxation time of the UHMWPE chains and subsequent residual stress removal. Furthermore, the scale of strip-like melting defects gradually deceased with the increase of residence time. When the residence time reaches 60 s, no explicit melting defects were observed from SEM images as indicated in Figure 1e. The observed morphology variation indicates that efficient welding occurs within a rather short time.

In order to assess the remaining porosity of the UHMWPE-nascent powders and various extruded UHMWPE samples, the density values acquired from the direct tests were compared with those values determined by the DSC crystal ratio according to the Equation (1):

(1) $$\rho =X_{c}\left(\rho_{c}-\rho_{a}\right)+\rho_{a}$$

where *ρ_a_* (0.855 g/cm^3^) and *ρ_c_* (1.000 g/cm^3^) represent the densities of the amorphous and crystalline phases, respectively, and *X_c_* and *X_c_*_1_ denote the crystallinity of UHMWPE nascent powders and extruded UHMWPE samples, respectively, as summarized in Table S1. The density values of the UHMWPE-nascent powders and the UHMWPE samples under an elongation flow with different residence times are shown in Figure 1d. According to previous studies [13], the difference of the densities from the direct test and the estimation from the DSC analysis is indicative of the remaining porosity of the samples. The measured density of UHMWPE nascent powders is 0.441 g/cm^3^, much lower than that calculated from DSC results (0.948 g/cm^3^), indicating the porous structures of nascent powders [14], as can be observed in Figure 1a. For the extruded samples prepared by the ERE, the density increases to 0.861 g/cm^3^, and the density gap variation between the two different methods reduces significantly to 0.058, owing to the compressive stress and continuous elongational flow generated by the ERE. However, the remaining porosity still exists, which is consistent with the SEM observation (Figure 1b). With increasing residence time, the density difference gradually reduces. For the extruded sample with the residence time of 20 s, the measured density of the UHMWPE-nascent powders is almost the same as that calculated from DSC results, suggesting no remaining porosity in the UHMWPE matrix. Interestingly, when we prolong the residence time over 20 s, the density values of UHMWPE samples are slightly higher than those calculated from DSC analysis. The density calculated from the DSC result is derived mainly based on a two-phase model. This model ignores the change of the third phase (intermediate phase), which is non-crystalline but an assembly of chains mainly of the trans conformation, thus leading to the underestimated density values [2].

### 3.2. Raman Mapping

**Figure 2:**
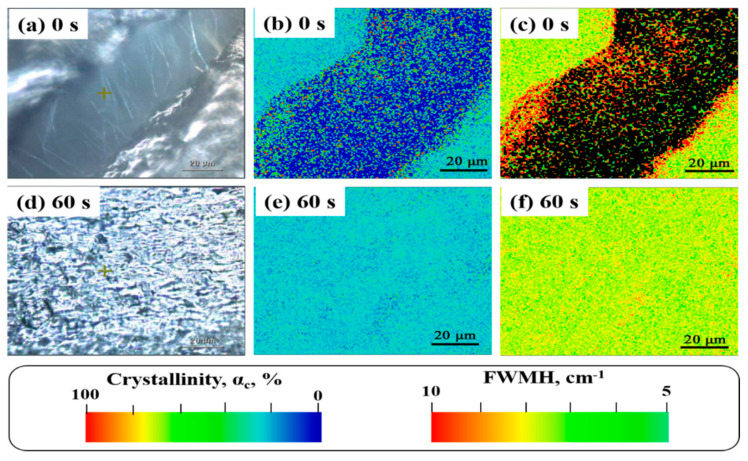
Extruded UHMWPE samples with different residence time of 0 s and 60 s. (**a**,**d**) Optical micrograph; (**b**,**e**) crystallinity distribution of Raman mapping; (**c**,**f**) FWMH (at 1130 cm^−1^) distribution of Raman mapping.

Figure 2 shows the optical micrographs and Raman mapping results for the extruded samples with the residence time from 0 s to 60 s while the corresponding Raman spectra are given in Figure S3. As can be seen from Figure 2a, the extruded sample with the residence time of 0 s showed a strip-like melting defect connected by fibrils in the optical microscopy while increased residence time of 60 s results in almost no melting effects as coincided with the above SEM observation. Raman spectra in Table S2 show several characteristic raman peaks, the raman band located around 1414 cm^−1^ is characteristic of the orthorhombic crystalline phase as well as other possible crystal polymorphisms. The band at 1080 cm^−l^ can be regarded as a stretching of the C–C skeleton in different types of gauche structures. The intensity of the 1130 cm^−l^ band can be associated with all-trans C–C bonds, independent of whether these are located in the crystalline or amorphous phase. The abovementioned characteristic peaks can be used as parameters for the crystalline phase, the amorphous phase and the intermediate phase [2]. Based on the Raman results, the values of crystalline phase (α_c_), amorphous phase (α_a_), and intermediate phase (α_b_) are calculated to be 42.18%, 23.44%, and 34.4%, respectively, following the literature-reported method (see detailed calculation details in supporting information). With increased residence time, the values of α_c_ gradually increase, since the increased entanglement hurdles the chain organization and recrystallization after melting. This variation tendency is found to be in agreement with the crystallinity obtained from DSC results, as shown in Table S3. In addition, the full-width at half-maximum (FWHM) at the 1130 cm^−1^ band was also calculated to examine the residual strain in the UHMWPE matrix [15]. Figure 2c,f shows the FWMH (at 1130 cm^−1^) distribution of Raman mapping. Clearly, the value of the FWHM is the largest for the extrude sample with the residence time of 0 s and increased residence time leads to gradually decreased FWHM. 

### 3.3. Polarized Raman Spectra

More useful information can be acquired by polarized Raman spectroscopy with its multiple uses in the vibrational modes that determine the molecular orientations of crystalline and amorphous phases. Herein, the Herman’s orientation function (ƒ) was used to examine the orientation behavior of the UHMWPE molecular chains, and can be defined as follows:

(2) $$f=\frac{<3{cos}^{2}\theta -1>}{2}=\frac{R-1}{R+2}\times \frac{2}{3{cos}^{2}\gamma -1}$$

where *θ* is the angle between the reference axis (based on the *x*-axis level) and the molecular chain axis, and γ is the angle between the transition dipole moment and the molecular chain axis. R is the dichroic ratio, which is defined as the ratio between parallel and perpendicular polarized intensities [16,17]. The 1130 cm^−1^ peak under an assumption of γ = 0° (parallel dichroism) was used for the calculation of ƒ. Depending on the molecular chain orientation, R can range from 0 to ∞, and ƒ is between −0.5 and 1. When the molecular chain axis corresponds perfectly to the reference axis, ƒ gives the maximum value, while imperfect correspondence leads to a smaller value. 

**Figure 3:**
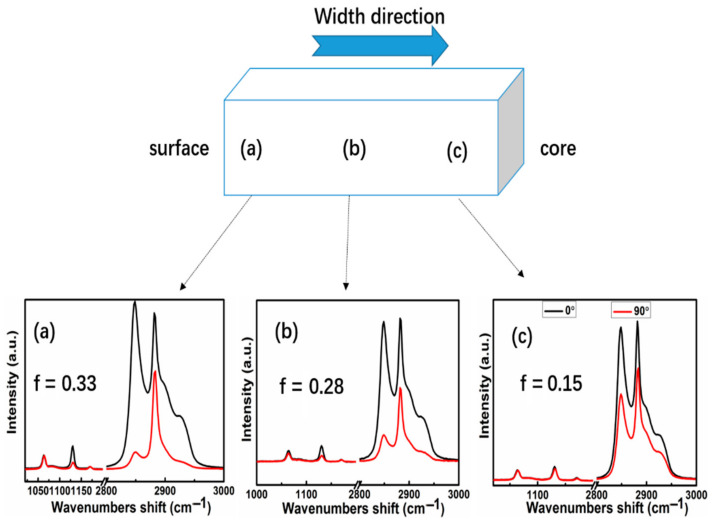
Polarized raman spectra of the UHMWPE samples along the transverse direction with 0 s residence time (**a**–**c**).

Figure 3 shows the polarized Raman spectra at different positions (region a, b, c) along the transverse direction of the extruded samples under a consecutive elongational flow with the residence time of 0 s of at 180 °C. In three regions (a, b, c), the polarized Raman spectra at 0°and 90° modes of the extruded sample exhibit a marked difference and the f value (0.15) at the core region c is much lower than that acquired at the surface region a (0.33), which indicates a strong orientation effect. The orientation degree of molecular chains reaches the highest level at surfaces. This result is different from the polarized Raman results in the case of the shear flow that exhibits little difference for 0° and 90° polarized modes as demonstrated in literature [17].

The Herman’s orientation function (ƒ) of the UHMWPE samples with surfaces under a consecutive elongational flow with different residence times was also determined, as shown in Figure S4**.** The results show that the molecular chains are oriented for all extruded samples. The obtained sample with no residence time exhibits the maximal ƒ value compared to other extruded samples. With increasing residence time, the values of ƒ gradually decrease due to the relaxation of UHMWPE chains. Undoubtedly, these chain motions enable UHMWPE molecules to relax their stresses or nonequilibrium conformations, eventually reducing the residual stress and orientation degrees of the extruded samples [18].

### 3.4. Chain Diffusion

**Figure 4:**
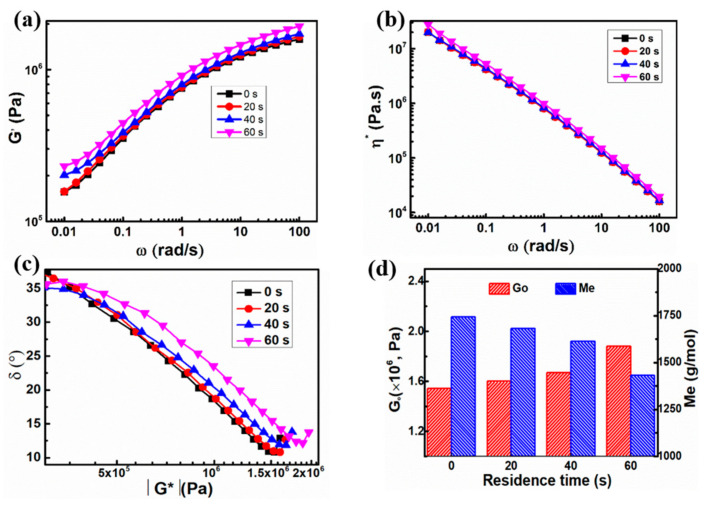
Frequency sweep of the UHMWPE samples prepared under the elongational flow for 0 s to 60 s residence time: (**a**) storage modulus; (**b**) complex viscosity; (**c**) van Gurp-Palmen (vGP) plot; (**d**) $G_{N}^{0}$ and *M_e_*.

To investigate the chain inter-diffusion through particle welding, the dynamic shear oscillatory measurements are performed. The results of the storage modulus (G’) and complex viscosity (η*) as a function of angular frequency for the UHMWPE samples processed at 180 °C are depicted in Figure 4a,b. It can be seen that the G’ values increase with increasing frequency because the probing time in the experiment is too short for the UHMWPE chains to relax [19]. Meanwhile, Figure 4b presents a linearly decreased η* with increased frequency for all the samples, demonstrating a non-Newtonian response. Moreover, with increased residence time, the G’ values of the UHMWPE samples at low frequency show a gradual increase, suggesting the increasing entanglement density [20]. The η* values at low frequency present a slight increase with increasing residence time, suggesting the increased entanglement density with no obvious molecular weight decrease. 

Considering that it is difficult to observe the plateau modulus ($G_{N}^{0}$) in the plots of storage modulus versus frequency in Figure 4a, the plateau modulus ($G_{N}^{0}$) is obtained by van Gurp-Palmen (vGP) plot [21], and it is estimated by the following formula:

G_0_ = G*(ω)_δ__→min_ (3)

where δ is phase angle and G* is complex modulus.

The relationship between the characteristic value of $G_{N}^{0}$ and the critical molecular weight between entanglement points *M_e_*, which is commonly given by Doi and Edwards, is built based on the theory of rubber elasticity as follows:

(4) $$M_{e}=\frac{K\rho \;R\;T}{G_{N}^{0}}$$

n = *M_η_/M_e_* (5)

where K is the numerical factor as 4/5, *ρ* is the density of molten PE (0.855 g/cm^3^), R is the ideal gas constant, and T is the absolute temperature. 

The calculated $G_{N}^{0}$ and *M_e_* values are collected in Figure 4d. As can be seen, with the increase of the residence time, the $G_{N}^{0}$ values increase, whereas the *M_e_* values reduce. For the UHMWPE sample with the residence time of 0 s, the *M_e_* value is 1745 g/mol. When the residence time reaches 60 s, the *M_e_* value was reduced to 1431 g/mol, which is much lower than that obtained from the molding process (4404 g/mol) and quite close to the commonly accepted *M_e_* of 1250 g/mol in rheology. Thus, it is concluded that increasing residence by a short time can enhance the chain mobility of the UHMWPE long chain and accelerate the diffusion of long polymer chains from one UHMWPE chain to another. This increased entangled density could behave as physical crosslinks to enhance the wear resistance.

### 3.5. Molecular Weight

**Figure 5:**
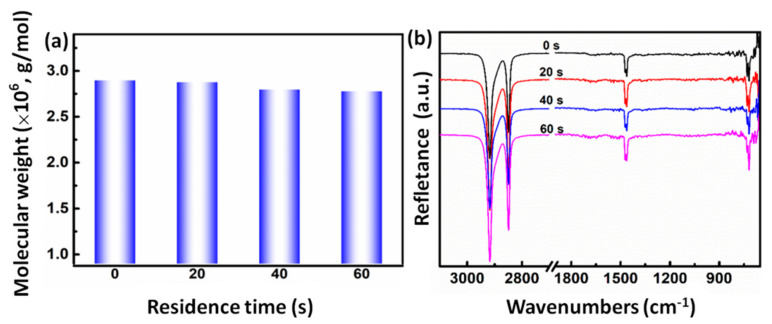
Molecular weight (**a**) and Infrared spectra (**b**) of the UHMWPE samples under a consecutive elongational flow for 0 to 60 s residence time.

Generally, molecular weight plays a key role on the rheological and mechanical properties. Thus, viscosity measurements and infrared spectra are carried out to further understand the influence of the residence time on molecular weight. As presented in Figure 5, with the increasing residence time of the extruded samples, the molecular weight of the UHMWPE exhibits no marked change, which is consistent with the above rheology results. Figure 5b exhibits the infrared spectra of the UHMWPE samples processed with various residence time at 180 °C. The peak at around 1720 cm^−1^, attributed to the carbonyl peak from the oxidative degradation, cannot be detected with the increasing residence time of the extruded samples, indicating less oxidation occurs during such processing [22]. All the above results indicate that the extremely low thermal oxidative degradation occurs with the increase of residence time.

Actually, the processing efficiency for the novel ERE method is much higher than that of the conventional method based on shear flow, such as sintering molding, and single screw extrusion molding. In addition, such new processing techniques processed at lower processing temperatures could largely maintain the molecular weight of the UHMWPE. Consequently, the cost of the novel ERE process is distinctly lower than those of commercial techniques, which is greatly helpful for the development of UHMWPE processing techniques.

### 3.6. Diffused Mechanism Discussion

Traditionally, the diffusion of long chain polymers in the rubbery state is depicted by the reptation theory [23]. The reptation time of UHMWPE with a molecular weight of 3.00 × 10^6^ g/mol at 180 °C is more than 12 h owing to its very high molecular weight. Thus, diffusion defects remain even after the 2 h sintering process time [13]. However, our findings show that UHMWPE molecular chains enable the chain diffusion and entanglement in much shorter time by regulating residence time under a CEF. Such phenomenon was also observed in the case of the sintering of nascent powder. The melting explosion phenomenon was demonstrated to occur for samples with various molecular weights; the higher the molecular weight is, the greater the efficiency would be [13]. These behaviors can be described by the so-called melting explosion process via fast sideway motions of the chains involving the proposed cooperative rouse motions [24]. However, sintering processing is still time-consuming, with the high risk of thermal degradation of UHMWPE.

The main issue is the ability for the chains to diffuse across the interface between powders to obtain an extruded material. The reptation model is used for UHMWPE to evaluate the diffusion at the interface [24] as follows:

(6) $$t_{rep}=\tau_{1}\frac{N^{3}}{N_{e}}$$

where *N* is the number of monomer units per chain, *N_e_* is the number of monomer units between entanglements, and *τ*_1_ is the microscopic time of monomer motion. This time is around 10^−10^ s and just above the melting temperature for PE. Wool and his coworkers have calculated the so-called average interpenetration distance at a polymer–polymer interface in order to model polymer welding based on the reputation theory as follows:

(7) $$\chi \left(t\right)=R_{g}\tau (\frac{t}{t_{rep}}{)}^{1/4}$$

where *R_g_* is the radius of gyration given as follows:

(8) $$R_{g}=b_{0}\sqrt{C_{\infty }\frac{N}{6}}$$

where *b*_0_ is the length bond and *C*_∞_ (6 for PE) is the characteristic ratio representing the flexibility of the chain using *N* = 280,000 and *N_e_* = 89. The calculated UHMWPE reptation time is more than 12 h due to the high *N* [13].

**Figure 6:**
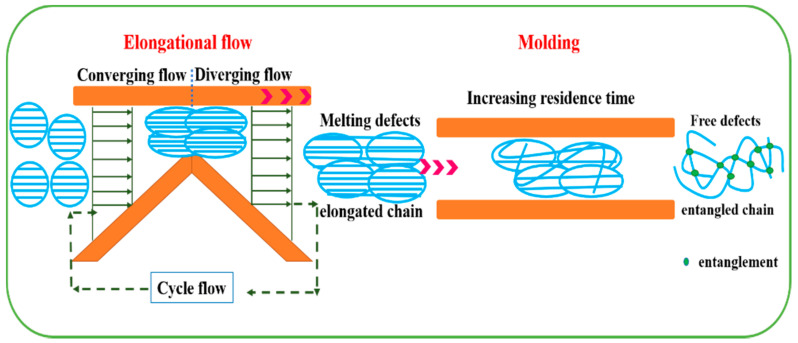
Schematic diagram of UHMWPE-nascent powders under an elongation flow with different residence time [18].

Doucet et al. [9] investigated the sintering mechanism by high velocity compaction of UHMWPE-nascent powders. They found that the mechanical properties of UHMWPE were assigned to the interface strength of the welded powders in relation to the kinetics of interfacial chain diffusion. However, this process is obtained by partial melting and subsequent recrystallization that enable wetting of nascent powders. In our study, the schematic diagram of the processing of the UHMWPE-nascent powders under an elongation flow with increasing residence time is presented in Figure 6. When the UHMWPE samples are processed by the ERE, UHMWPE-nascent powders are compressed dramatically and the long polymer chains of UHMWPE are prone to orientate under a consecutive volume elongational deformation, resulting in the higher residual stress for the extruded sample. Such nonequilibrium structure can drastically increases the volume of the UHMWPE after melting to recover their equilibrium configuration and the polymeric chains tend to entangle within a short time, which is called melting explosion proposed by De Gennes. During the melting explosion process, the intertwining of chains from neighbor particles by sideway motions of initially disentangled chain loops is much fatter than the reptation of chains by both ends along their tube. As a result, the enhanced diffusion of the chain segments can be achieved by increasing the residence time within a relatively short time regardless of molecular weight, resulting in the more uniform morphology and higher entanglement with lower crystallinity. The main mechanism of the fast chain reentanglement and particle welding for such long molecular chain is the entropy-driven melting explosion over distances much larger than the chain length between entanglements.

## 4. Conclusions

In conclusion, we have demonstrated that UHMWPE without any processing aids can be processed at 180 °C—far below conventional processing temperature by the ERE and probed the effect of residence time of UHMWPE under a consecutive elongational flow on the morphology, rheological behavior and orientation of UHMWPE molecular chains. It is found that the UHMWPE-nascent powders are compressed drastically, and the long polymer chains of UHMWPE are apt to orientate under a consecutive volume elongational deformation. Thus, the obtained extruded samples display a higher residual stress if no residence time is applied. Increasing the residence time within a relatively short time (up to 60 s) enables the polymeric chain relaxation and total removal of the residual stress, eventually generating a defect-free morphology and higher entanglement density of the UHMWPE with lower crystallinity and low thermal oxidative degradation. Such fast chain entanglement and particle welding induced by the residence can be explained by the entropy-driven melting explosion over distances much larger than the chain length between entanglements. This work illustrates the huge potential of the ERE technique to process UHMWPE materials with high efficiency.

## Data Availability

The data presented in this study are available on request from the corresponding author.

## Supplementary Materials

The following are available online at https://www.mdpi.com/article/10.3390/polym13132192/s1, Figure S1: Eccentric rotor extruder, Figure S2: Photograph of UHMWPE samples under elongation flow samples at 180 °C with 0 s, 20 s, 40 s, 60 s residence time, Figure S3: Raman spectra of the UHMWPE sample under a consecutive elongational flow with 0 s, 20 s, 40 s, 60 s residence time, Figure S4: Hermans orientation function (ƒ) of the UHMWPE samples at surface under a consecutive elongational flow from 0 to 60 s seconds residence time, Table S1: DSC analysis result of the UHMWPE nascent powder and the UHMWPE sample produced at 180 °C with different residence time, Table S2: Assignments of the main Raman bands of UHMWPE in the C–C), CH2) and CH2) ranges (indicated as region I, II and III, respectively), adopted from Ref. 2, Table S3: Raman and DSC analysis results of the UHMWPE samples prepared at 180 °C with different residence time, Table S4: Kinetics values for the UHMWPE chain diffusion according to the reptation theory.
