# Supplementary material for: Enhancing Chain Mobility of Ultrahigh Molecular Weight Polyethylene by Regulating Residence Time under a Consecutive Elongational Flow for Improved Processability
Source: Polymers (Basel). 2021 Jun 30;13(13):2192. doi: 10.3390/polym13132192 (PMC8271948; doi:10.3390/polym13132192)
Supplement: Supplementary file 1 [file polymers-13-02192-s001.zip › polymers-1251235-supplementary.pdf]

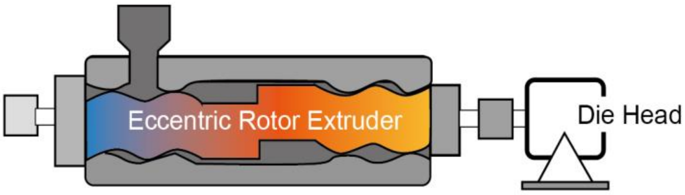

Figure S1. Eccentric rotor extruder

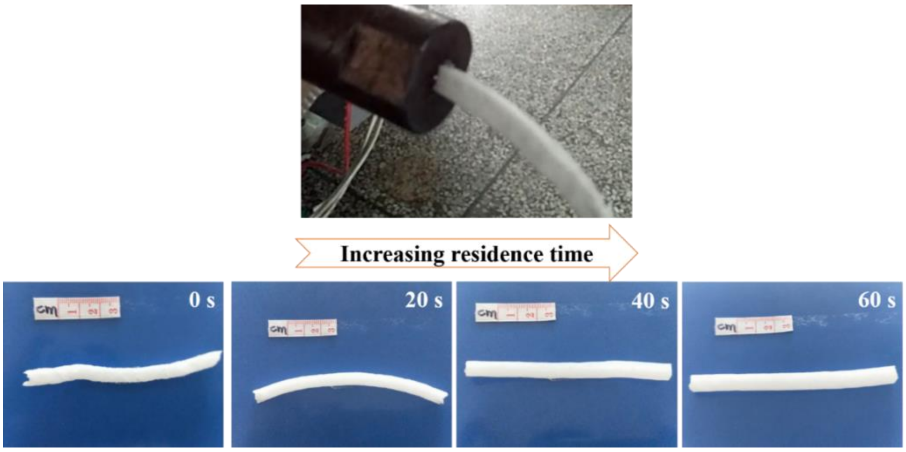

Figure S2. Photograph of UHMWPE samples under elongation flow samples at 180 °C with 0s.20s.40s.60s residence time.

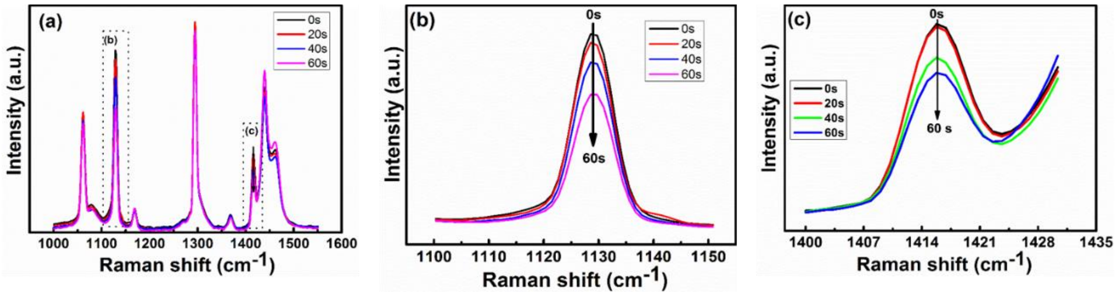

Figure S3. Raman spectra of the UHMWPE sample under a consecutive elongational flow with 0s.20s.40s.60s residence time.

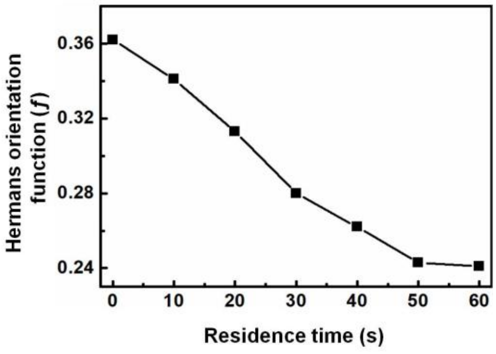

Figure S4. Hermans orientation function (f) of the UHMWPE samples at surface under a consecutive elongational flow from 0 to 60s seconds residence time.

Table S1. DSC analysis result of the UHMWPE nascent powder and the UHMWPE sample produced at 180 °C with different residence time.

Sample	T_{m1} (°C)	$\triangle H_{m1}$ (J/g)	X_{c1} (%)	T_{m2} (°C)	$\triangle H_{m2}$ (J/g)	X_{c2} (%)	T_c (°C)
Nascent powder [1]	143.2	185.9	64.1	136.0	137.0	47.2	116.7
0 s	137.5	128.4	44.3	131.2	131.2	45.2	116.86
20 s	139.2	118.5	40.9	138.5	118.6	40.9	116.21
40 s	139.23	113.5	39.2	139.6	116.4	40.1	114.7
60 s	139.1	94.67	32.6	139.5	96.4	33.2	115.32

Note: “1” and “2” stand for the first heating run and the second heating run respectively.
[1]: The data is adopted from Ref. 1.

Table S2. Assignments of the main Raman bands of UHMWPE in the ν(C-C), τ(CH_2) and δ(CH_2) ranges (indicated as region I, II and III, respectively), adopted from Ref. 2

**Raman shift(cm^{-1})**	**Mode**	**Phase**
1060	ν_{as} (C-C)	Crystalline + amorphous trans
1080	ν(C-C)	Amorphous gauche
1130	ν_s(C-C)	Crystalline + amorphous trans
1170	ν_ρ (CH_2)	Crystalline + amorphous trans
1295	τ(CH_2)	Crystalline
1305	τ(CH_2)	Amorphous gauche
1416	δ(CH_2)	Crystalline
1440	δ(CH_2)<br>Fermi resonance	Amorphous trans (interphase)
1460	δ(CH_2)	Amorphous
2848	ν_s (CH_2)	
2881	ν_{as} (CH_2)	

δ = bending ; τ = twisting ; ν = stretching ;

ρ = rocking ; s = symmetric ; as = antisymmetric.

Note: the data in Tab. S2 is adopted from Ref. 2.

Table S3. Raman and DSC analysis results of the UHMWPE samples prepared at 180 °C with different residence time.

Sample (s)	DSC-X_{c1} (%)	Raman-α_c (%)	Raman-α_b (%)	Raman-α_a (%)
0	45.2	42.18	23.44	34.4
20	40.9	39.9	28.2	31.9
40	40.1	36.6	27.5	35.9
60	33.2	31.8	28.1	41.2

Table S4. Kinetics values for the UHMWPE chain diffusion according to the reptation theory

Mw(g/mol)	τ_1	N	Ne	trep	Rg	t(x=Rg)
1.0×10^{6}	1.0×10^{-10}	3.57×10^{4}	89.0	5.12×10^{1}	2.08×10^{-7}	5.12×10^{1}
2.0×10^{6}	1.0×10^{-10}	7.14×10^{4}	89.0	4.09×10^{2}	2.94×10^{-7}	4.09×10^{2}
3.0×10^{6}	1.0×10^{-10}	1.07×10^{4}	89.0	1.38×10^{3}	3.60×10^{-7}	1.38×10^{3}
4.0×10^{6}	1.0×10^{-10}	1.43×10^{4}	89.0	3.28×10^{3}	4.16×10^{-7}	3.28×10^{3}
5.0×10^{6}	1.0×10^{-10}	1.79×10^{4}	89.0	6.40×10^{3}	4.65×10^{-7}	6.40×10^{3}
6.0×10^{6}	1.0×10^{-10}	2.14×10^{4}	89.0	1.11×10^{4}	5.09×10^{-7}	1.11×10^{4}
7.0×10^{6}	1.0×10^{-10}	2.50×10^{4}	89.0	1.76×10^{4}	5.50×10^{-7}	1.76×10^{4}
9.0×10^{6}	1.0×10^{-10}	2.80×10^{4}	89.0	2.47×10^{4}	5.82×10^{-7}	2.47×10^{4}

Note: *N* is the number of monomer units per chain; *Ne* is the number of monomer units between entanglements; τ_1 is the microscopic time of monomer motion.

References

1. Cao, C.L.; Chen, X.C.; Wang, J.X.; Lin, Y.; Guo, Y.Y.; Qian, Q.R.; Chen, Q.H.; Feng, Y.H.; Yu, D.S.; Chen, X.D. Structure and properties of ultrahigh molecular weight polyethylene processed under a consecutive elongational flow. *J Polym Res*. **2018**, *25*, 16-25.
2. Pezzotti, G. Raman spectroscopy of biomedical polyethylenes. *Acta Biomater*. **2017**, *55*, 28-99.
